# Evaluation of Lumbar Intervertebral Disc and Facet Joint Degeneration Using Histogram Analysis of T2 and T2* Values

**DOI:** 10.1002/jsp2.70141

**Published:** 2025-11-26

**Authors:** Xiaoqing Liang, Yitong Li, Bowen Hou, Yan Xiong, John Morelli, Weiyin Vivian Liu, Xiaoming Li

**Affiliations:** ^1^ Department of Radiology Tongji Hospital, Tongji Medical College, Huazhong University of Science and Technology Wuhan China; ^2^ Department of Magnetic Resonance Imaging The First Affiliated Hospital of Zhengzhou University Zhengzhou China; ^3^ Russell H. Morgan Department of Radiology and Radiological Science Johns Hopkins University School of Medicine Baltimore Maryland USA; ^4^ MR Research, GE Healthcare Beijing China

**Keywords:** degeneration, facet joint, histogram analysis, intervertebral disc, T2 mapping, T2* mapping

## Abstract

**Background:**

Lumbar facet joint (LFJ) and intervertebral disc (IVD) degeneration are the common causes of low back pain. The aim of this study is to explore the feasibility of histogram analysis of T2 and T2* values on grading LFJ and IVD degeneration and to examine the correlation between the LFJ and IVD in the degenerative process.

**Methods:**

420 IVDs and 840 LFJs of 87 subjects were examined using T2WI, T2 and T2* mapping. All IVDs and LFJs were classified, respectively, according to the Pfirrmann and Weishaupt grade and grouped by patient age. Histogram‐derived parameters based on T2 (T2‐HPs) and T2* (T2*‐HPs) values of IVDs and LFJs were compared among the different groups.

**Results:**

The interobserver agreement for Pfirrmann grade was good (*κ* = 0.732), and moderate for Weishaupt grading (*κ* = 0.474). For patients under 39 years old, the degeneration incidence (DI) of LFJ was higher than IVD (*χ* ^2^ = 16.436, *p* < 0.001; *χ* ^2^ = 5.210, *p* = 0.022). In the 50–59 and 60–69 years groups, the DI of LFJ was statistically significantly lower than that of IVD (*χ* ^2^ = 14.915, *p* < 0.001; *χ* ^2^ = 13.174, *p* < 0.001)*.* The interobserver reliability of histogram parameters for IVDs was good to excellent with ICCs ranging from 0.825 to 0.985, and poor to excellent for LFJs (0.302–0.945). All T2‐HPs and T2*‐HPs had the ability to distinguish normal IVDs from abnormal discs, with the AUC varying from 0.562 to 0.824. For T2‐HPs, only SD and Entropy can not distinguish normal (Weishaupt grades 0 and 1) and abnormal (grades 2 and 3) LFJs, and all other parameters can distinguish them, with AUC changing from 0.551 to 0.615. For T2*‐HPs, only Mean and Entropy were reliable for identifying normal and abnormal LFJs with low AUC (0.572, 0.540, respectively).

**Conclusions:**

Histogram analysis of T2/T2* values is feasible for detecting IVD degeneration, but the feasibility of grading LFJ is still controversial. The DI of LFJ is higher than that of IVD under 39 years old, challenging the commonly accepted paradigm of the degenerative process beginning at the IVDs.

## Graphical abstract

Histogram Assessment of Lumbar Intervertebral Disc and Facet Joint Degeneration.

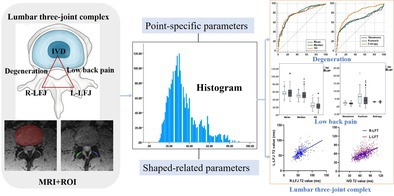

## 1 Introduction

Low back pain (LBP) is a serious health problem among adolescents and especially in middle‐aged and elderly populations, leading to a major economic burden on health care systems worldwide [1, 2, 3]. LBP may arise from different spinal structural abnormalities. Lumbar intervertebral disc (IVD) degeneration is recognized as the leading cause of LBP [1, 4], while lumbar facet joint (LFJ) degeneration is another source of pain accounting for 15%–45% of LBP [2, 3, 5].

At each lumbar segment of the spine, the anterior IVD and posterior two LFJs form a three‐joint complex, allowing the vertebral bodies to transfer loads, bend flexibly, and maintain stability of the spine [6, 7]. IVDs and LFJs are the primary and important load‐bearing structures in the spine, and any degenerative abnormality in them may lead to spinal instability and LBP [8, 9, 10]. The main degenerative form of LFJs is osteoarthritis with articular cartilage matrix loss, while IVD degeneration is characterized by loss of water, hydrated proteoglycan and collagen matrix [11, 12, 13]. At present, it is still unclear which structure, LFJ or IVD, typically degenerates first, because all individual degenerative changes of the lumbar segments have been observed separately or in some combination in previous studies [14, 15, 16, 17]. Moreover, the details of the interaction between the disc and the two facet joints in the three‐joint complex are also unclear.

Numerous studies [4, 18, 19] have explored the biochemical changes of disc degeneration and its relevance in LBP using various quantitative magnetic resonance imaging (qMRI) techniques. However, due to the complicated anatomical position, the differing orientations of the articular surface, and the tiny size of LFJ, it has been studied infrequently and often improperly evaluated clinically [11]. T2 mapping and T2* mapping have been recognized as promising noninvasive tools for the quantitative assessment of articular cartilage in recent years [12, 20]. Many studies have confirmed that T2/T2* relaxation times correlate with water and glycosaminoglycan content of IVDs [21, 22], Pfirrmann grading [12, 20], and LBP [12]. Additionally, compared with other quantitative sequences (e.g., T1ρ mapping and Chemical Exchange Saturation Transfer), T2 and T2* mapping exhibit higher technical maturity, stronger adaptability to hardware and scanning conditions, as well as greater stability and repeatability of the measurement results [23, 24, 25]. Additionally, T2 and T2* mapping require shorter scan times, which reduces patient discomfort and motion artifacts caused by prolonged lying, decreases, and thus offers high clinical applicability [25]. Therefore, this study attempts to use T2/T2* mapping to assess the aging sequence and relationships of IVDs and LFJs, thereby aiding in the diagnosis and eventual treatment of LBP.

Histogram analysis is a popular approach for obtaining quantitative measurements and characterizing microstructural heterogeneity within a given region of interest (ROI) by obtaining metrics that assess the frequency and arrangement of single‐voxel [26, 27]. It has the advantage of not relying on image grayscales of different MR systems and subjective judgment of the diagnostic doctors, making for the shortcoming that the mean value cannot reflect the spatial distribution [28, 29]. Histogram analysis has achieved significant progress in assessing the heterogeneity of organs and lesions, such as evaluating the activity of lesions or organ functions [28, 30, 31], distinguishing benign and malignant tumors [32], and predicting grade, histological type and prognosis of tumors, etc. [26, 27, 33, 34]. To the best of our knowledge, some articles have explored the feasibility of MRI histogram analysis in disc degeneration [35, 36, 37, 38], but no studies have been performed to evaluate LFJ degeneration using histogram analysis.

Therefore, the aim of this study was to examine the feasibility of T2 and T2* value histogram analysis for detection and gradation of IVD and LFJ degeneration and to explore the correlation between IVD and LFJ degeneration.

## 2 Materials and Methods

### 2.1 Study Population

This study was approved by the institutional review board of our hospital. 87 subjects (47/40 women/men) with a mean age of 41.66 ± 13.52 years (range 22–69 years) who underwent both morphological MRI (sagittal and axial T2WI) and biochemical MRI (axial T2 and T2* mapping) at our hospital between May 2018 and November 2021 were retrospectively studied. 87 subjects were divided into two groups: (1) LBP group: included 66 patients with chronic nonspecific LBP; the inclusion criteria were adult patients with chronic LBP, which was defined as back pain lasting for more than 3 months; (2) normal control (NC) group: included 21 normal subjects. Exclusion criteria for all subjects were exact pathology (including injury, infection, tumor, ankylosing spondylitis, spinal deformity, and previous spine surgery), dysfunction of lower extremity (radicular syndromes), systemic disease (metabolic or hematological) and any MRI sequence deficiency.

### 2.2 MR Imaging Protocol

All MR examinations were performed on a 3.0T MR system (GE Discovery MR 750; GE Healthcare, Waukesha, WI, USA) with a dedicated eight‐channel spine coil. The subjects were imaged in the supine position with five lumbar spinal segments (each with 5 IVDs and 10 LFJs).

Axial and sagittal T2‐weighted fast‐spin‐echo (FSE) sequences were used for the morphological evaluation of the IVDs and LFJs (Axial: TR/TE 2000 ms/99.5 ms, thickness/spacing 4 mm/0.5 mm, field of view (FOV) 200 mm, examination time 2:16 min; sagittal: TR/TE 2500 ms/120 ms, thickness/spacing 4 mm/0.5 mm, FOV = 320 mm, examination time 0:55 min).

Axial T2 and T2* mapping were performed using multi‐echo spin and gradient echo sequences (T2 mapping: TR/TEs = 1600/7.55; 15.10; 22.66; 30.21; 37.76; 45.31; 52.86; 60.42 ms, FOV 200 mm, thickness/spacing 2.2 mm/1 mm, flip angle 90°, and examination time 4:19 min; T2* mapping: TR/TEs = 150/2.75; 4.20; 5.66; 7.12; 8.57; 10.03; 11.48; 12.94; 14.40; 15.85; 17.31; 18.76; 20.22; 21.68; 23.13; 24.59 ms, FOV 200 mm, thickness/spacing 2.2 mm/1 mm, flip angle 30°, and examination time 2:03 min). Five axial slabs (each with 3 slices) were used to cover the five lumbar segments (L1/2 to L5/S1, a total of 15 slices) and aligned along the corresponding discs.

### 2.3 Image Analysis and Post‐Processing

All discs and facet joints were visually graded based on morphological MRI sequences, respectively according to the Pfirrmann grading [39] and the Weishaupt grading system [40] by two independent radiologists with more than 10 years of musculoskeletal MRI experience. Pfirrmann grades I and II were defined as “normal” and grades III to V as “abnormal” [11]. Similarly, Weishaupt grades 0 and 1 were defined as “normal” and grades 2–3 as “abnormal”. When there was disagreement on the grading opinions between two readers, the images were reviewed in consensus. The degeneration incidence (DI) in each group was calculated as: DI = Abnormal/Total.

**FIGURE 1:**
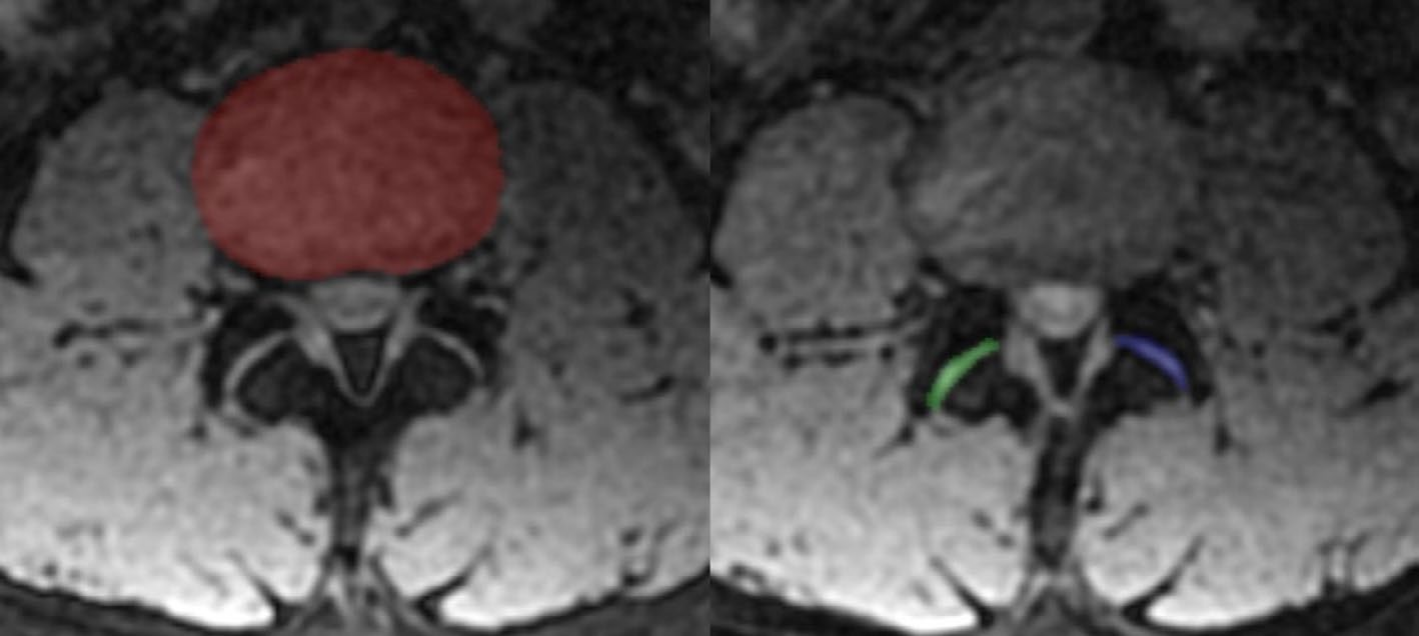
The ROIs of lumbar intervertebral discs (IVDs) and facet joints (LFJs). (a) ROIs of IVDs were drawn on the second of 3 consecutive T2/T2* mapping images slices (slice 2 of 3); (b) ROIs of LFJs were drawn on the third slice (slice 3 of 3) on both sides.

The ROIs for IVDs and LFJs were manually selected on the FireVoxel software (Department of Radiology, New York University, USA) by two other readers with 5 years of MRI experience. Axial T2 and T2* map images (DICOM format) were imported into FireVoxel. Each IVD was manually outlined only on the second slice of three consecutive images (slice 2 of 3) in order to avoid structural interference originating from the endplates, and each LFJ was outlined on the third slice [11, 12, 20] (Figure 1). Generally, an echo that best depicted the discs and facet joints is utilized to outline the ROIs. In this study, the echoes with the smallest TE for T2 and T2* mapping were selected. Then the software automatically generated T2 and T2* color maps and histogram‐derived parameters of T2 and T2* value (T2‐HPs and T2*‐HPs), including point‐specific (mean, median, 10th, 25th, 75th and 90th percentiles) and histogram shape‐related parameters (standard deviation, kurtosis, skewness, and entropy); the two sets of values were averaged for further statistical analysis.

### 2.4 Statistical Analysis

Statistical analysis was performed using SPSS 22.0 (SPSS Institute, Chicago, IL, USA) and MedCalc (MedCalc Software, Mariakerke, Belgium). *p* values < 0.05 were deemed statistically significant.

The Shapiro–Wilk test was used for normality, and the Levene test was used to analyze the homogeneity of variance. Independent *t*‐test and Chi‐square test were used to compare age and sex differences between the LBP group and the NC group respectively. Kappa statistics were used to assess the agreement of the Weishaupt and Pfirrmann grading results between two readers (*κ* > 0.80, excellent agreement; 0.80 ≥ *κ* > 0.60, good agreement; 0.60 ≥ *κ* > 0.40, moderate agreement; 0.40 ≥ *κ* > 0.20, fair agreement; and *κ* < 0.20, poor agreement) [41]. Chi‐square tests were used to compare the gender differences and the DI differences in different age groups, the DI differences between the IVDs and LFJs in the same age groups. Intraclass correlation coefficients (ICCs) with 95% confidence intervals (CIs) were calculated to evaluate the reproducibility of T2‐HPs and T2*‐HPs(ICC < 0.5, poor reliability; 0.5 ≤ ICC < 0.75, moderate reliability; 0.75 ≤ ICC < 0.9, good reliability; ICC > 0.9, excellent reliability) [42]. One‐way ANOVA and least‐significant difference (LSD) tests or Kruskal–Wallis H and pairwise comparison tests were used to examine the statistical significances of IVD/LFJ T2‐HPs and T2*‐HPs in the different Pfirrmann grading/Weishaupt grading groups. The Benjamini‐Hochberg method was applied to correct statistical significance and obtain adjusted *p*‐values (*p*.adj) for the issue of multiple comparisons, and a false discovery rate of 0.05 had been specified in advance. Pearson or Spearman coefficient tests were performed to assess the correlation between histogram‐derived parameters of IVD/LFJ and Pfirrmann grade/Weishaupt grade as well as the correlation of T2‐HPs and T2*‐HPs between the anterior IVD and the posterior bilateral LFJs in the three‐joint complex. Kendall's tau‐b correlation analysis was used to assess the correlation between the IVD Pfirrmann grades and the LFJ Weishaupt grades in the three‐joint complex. For the above correlation analysis, appropriate clustering analysis was performed based on the 5 lumbar intervertebral levels (L1/2 to L5/S1) to avoid artificially inflated correlation caused by samples derived from the same individual. The absolute value of the correlation coefficient *r* was used to assess the strength of the relationship (|*r*| ≥ 0.7, high correlation; 0.7 > |*r*| ≥ 0.4, moderate correlation; |*r*| < 0.4, low correlation). Receiver operating characteristic (ROC) curves analysis was used to determine the sensitivity and specificity of T2‐HPs and T2*‐HPs for identifying normal and abnormal IVDs/LFJs. Mann–Whitney *U* test was used to assess the difference in Pfirrmann/Weishaupt grading between the LBP group and the NC groups; *t* test or Mann–Whitney *U* test were used to compare the difference of T2‐HPs and T2*‐HPs between the two groups (the spinal segment with the highest Pfirrmann/Weishaupt grades in each individual were selected for comparison, and the closest to the caudal vertebrae were selected if there were multiple highest grades simultaneously).

## 3 Results

### 3.1 Subject Characteristics

LBP group included 66 patients with chronic nonspecific LBP (31/35 women/men, 23–69 years, mean age was 43.12 ± 14.02 years), and the NC group included 21 normal subjects (9/12 women/men, 22–58 years, mean age was 37.29 ± 11.28 years). There were no significant differences in age and sex between the two groups (*p* = 0.138; 0.742).

A total of 420 IVDs and 840 LFJs were investigated. Fifteen IVDs and adjacent thirty LFJs were excluded because of poor image quality or motion artifact. All subjects were divided into four groups based on age: 23 subjects (11/12 men/women) with 113 IVDs and 226 LFJs were divided into the 20–29 years group, 19 subjects (8/11 men/women) with 89 IVDs and 178 LFJs in the 30–39 years group, 17 subjects (8/9 men/women) with 85 IVDs and 170 LFJs in the 40–49 years group, 15 subjects (7/8 men/women) with 70 IVDs and 140 LFJs in the 50–59 years group, and 13 subjects (6/7 men/women) with 63 IVDs and 126 LFJs in the 60–69 years group. No significant difference was found in gender between different age groups (*χ* ^2^ = 0.157, *p* = 0.997).

### 3.2 Pfirrmann/Weishaupt Grading Results

Two radiologists had excellent intraobserver agreement on Pfirrmann grading of 420 IVDs (radiologist 1: *κ* = 0.811, *p* < 0.001; radiologist 2: *κ* = 0.803, *p* < 0.001) and good interobserver agreement (*κ* = 0.732, *p* < 0.001). 211 discs (50.24%) were graded as normal (67 as Pfirrmann I and 144 as Pfirrmann II) and 209 (49.76%) as abnormal (107 as Pfirrmann III, 87 as Pfirrmann IV and 15 as Pfirrmann V).

The intraobserver agreement (radiologist 1: *κ* = 0.524, *p* < 0.001; radiologist 2: *κ* = 0.518, *P* < 0.001) and interobserver agreement (*κ* = 0.474, *P* < 0.001) for Weishaupt grading of 840 LFJs was moderate. 431 (51.31%) LFJs were graded as normal (78 as grade 0 and 353 as grade 1) and 409 (48.69%) as abnormal (321 as grade 2 and 88 as grade 3).

### 3.3 The DI of IVD and LFJ With Age

**FIGURE 2:**
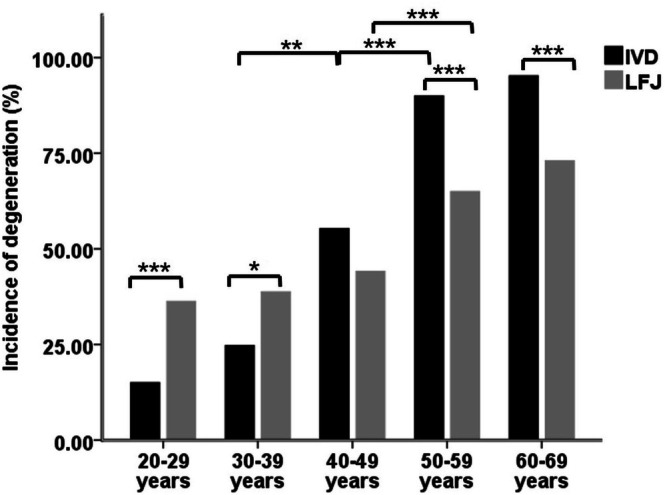
Degeneration incidence (DI) of IVDs and LFJs in different age groups. *p < 0.05, **p < 0.01, ***p < 0.001.

Figure 2 shows DI of IVD and LFJ in different age groups. The DI of both discs and facets increased with age; the DI of IVDs for five successive age groups was 15.04%, 24.72%, 55.29%, 90.00% and 95.24%; the DI of LFJs for those age groups was 36.28%, 38.76%, 44.12%, 65.00% and 73.02%. For IVDs, significant differences were found between the 30–39 and 40–49 age groups, and between the 40–49 and 50–59 age groups (*χ* ^2^ = 2.991, *p* = 0.084; *χ* ^2^ = 7.688, *p* = 0.006, *χ* ^2^ = 34.691, *p* < 0.001; *χ* ^2^ = 0.663, *p* = 0.415, successively). For LFJs, a significant difference was only found between the 40–49 year and 50–59 year age groups (*χ* ^2^ = 0.262, *p* = 0.609; *χ* ^2^ = 1.027, *p* = 0.311; *χ* ^2^ = 13.459, *p* < 0.001; *χ* ^2^ = 1.985, *p* = 0.159, successively). The DI of LFJ was higher than the IVD in both 20–29 years and 30–39 years groups, and the difference was statistically significant (*χ* ^2^ = 16.436, *p* < 0.001; *χ* ^2^ = 5.210, *p* = 0.022). In the 50–59 and 60–69 years groups, the DI of LFJ was statistically significantly lower than that of IVD (*χ* ^2^ = 14.915, *p* < 0.001; *χ* ^2^ = 13.174, *p* < 0.001). There was no statistical difference between IVD and LFJ DI in the 40–49 year group(*χ* ^2^ = 0.008, *p* = 0.929).

### 3.4 Histogram‐Derived Parameters of T2 and T2* Values of IVDs and LFJs

The interobserver reliability for the measurements of IVD T2‐HPs and T2*‐HPs was good to excellent with ICCs ranging from 0.825 to 0.985 and 95% CI from 0.792 to 0.988; the interobserver reliability for the measurements of LFJ T2‐HPs and T2*‐HPs varied from poor to excellent with ICCs ranging from 0.302 to 0.945 and 95% CI from 0.221 to 0.952 (Table 1).

**TABLE 1:** Interobserver reliability for histogram‐derived parameters.

	Intraclass correlation coefficient (95% confidence intervals)			
	T2‐HPs of IVDs	T2*‐HPs of IVDs	T2‐HPs of LFJs	T2*‐HPs of LFJs
Mean	0.972 (0.966, 0.977)	0.969 (0.963, 0.974)	0.906 (0.893, 0.918)	0.935 (0.926, 0.943)
SD	0.928 (0.914, 0.940)	0.975 (0.970, 0.979)	0.748 (0.711, 0.780)	0.746 (0.715, 0.775)
Median	0.960 (0.952, 0.967)	0.914 (0.897, 0.929)	0.945 (0.937, 0.952)	0.943 (0.935, 0.950)
5th	0.949 (0.938, 0.957)	0.914 (0.897, 0.929)	0.847 (0.825, 0.866)	0.822 (0.797, 0.845)
10th	0.959 (0.950, 0.966)	0.912 (0.895, 0.927)	0.872 (0.854, 0.888)	0.743 (0.706, 0.775)
25th	0.955 (0.946, 0.963)	0.887 (0.864, 0.905)	0.900 (0.885, 0.912)	0.910 (0.897, 0.922)
75th	0.895 (0.874, 0.912)	0.968 (0.961, 0.973)	0.867 (0.847, 0.883)	0.900 (0.885, 0.913)
90th	0.897 (0.877, 0.914)	0.985 (0.982, 0.988)	0.688 (0.643, 0.728)	0.828 (0.803, 0.850)
95th	0.890 (0.868, 0.908)	0.977 (0.973, 0.981)	0.690 (0.645, 0.729)	0.770 (0.737, 0.799)
Skewness	0.831 (0.795, 0.861)	0.970 (0.964, 0.976)	0.356 (0.263, 0.438)	0.466 (0.389, 0.543)
Kurtosis	0.825 (0.792, 0.854)	0.977 (0.972, 0.981)	0.302 (0.221, 0.406)	0.306 (0.206, 0.394)
Entropy	0.875 (0.850, 0896)	0.969 (0.962, 0.974)	0.677 (0.630, 0.718)	0.740 (0.708, 0.769)

Abbreviations: IVDs: intervertebral discs; LFJs: lumbar facet joints; T2*‐HPs: histogram‐derived parameters of T2* values; T2‐HPs: histogram‐derived parameters of T2 values.

**FIGURE 3:**
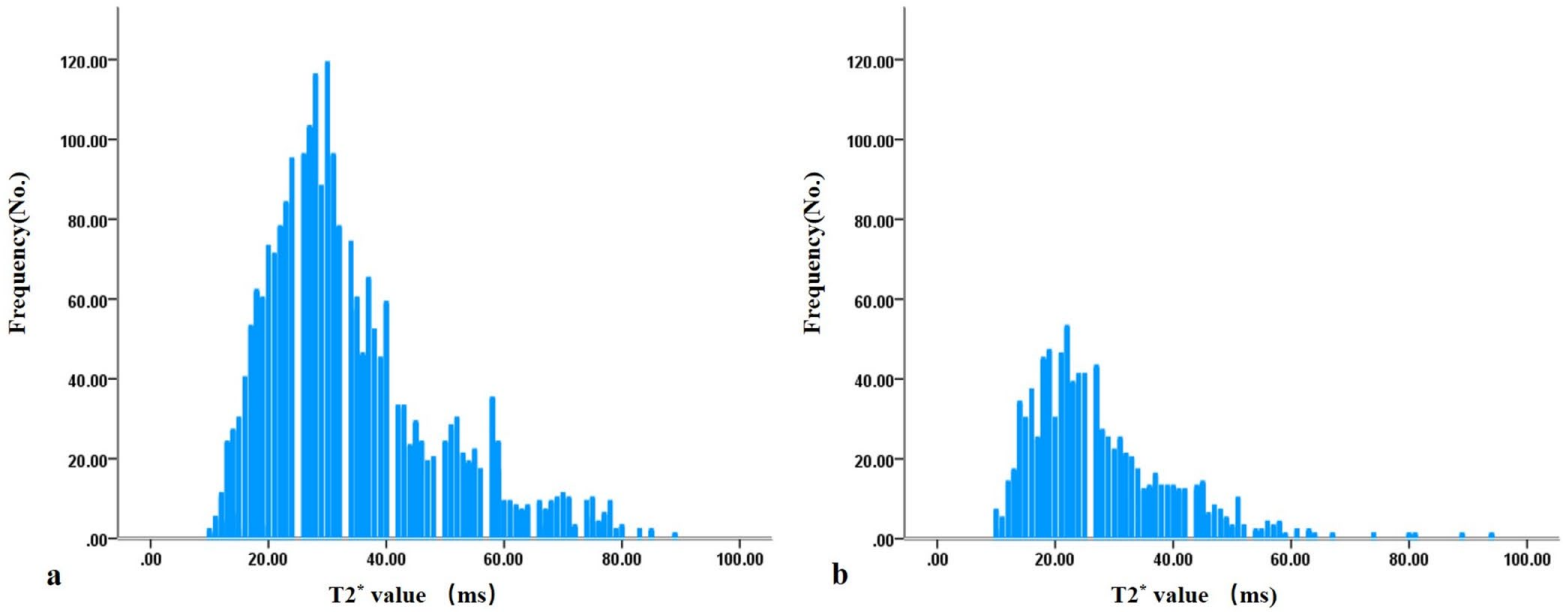
Histograms of T2* value pixel distribution for a normal intervertebral disc (Pfirrmann Grade I, Figure a) and a degenerated intervertebral disc (Pfirrmann Grade V, Figure b).

**FIGURE 4:**
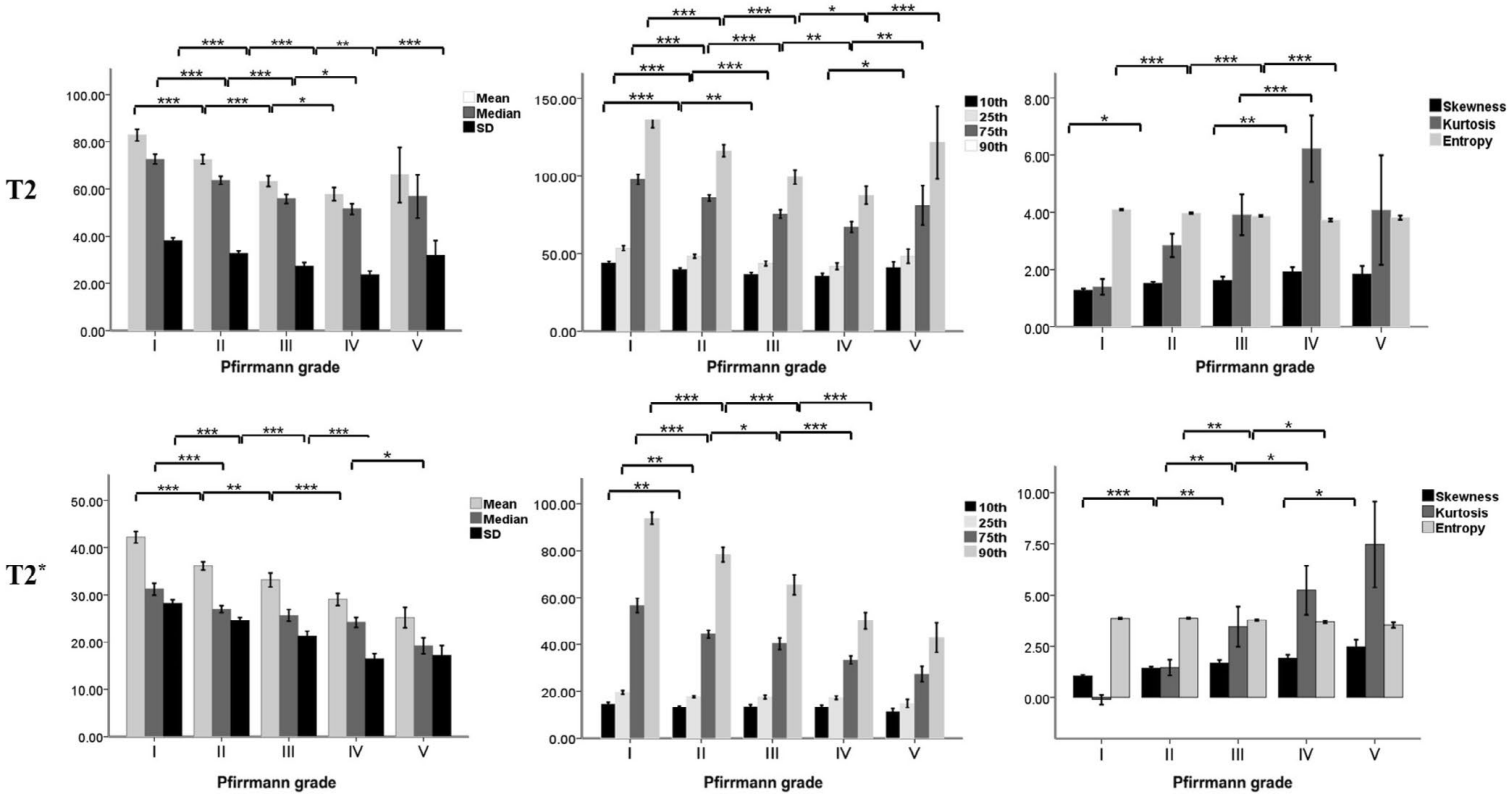
The T2‐HPs and T2*‐HPs of intervertebral discs with increasing Pfirrmann grade. T2‐HPs: histogram‐derived parameters of T2 values; T2*‐HPs: histogram‐derived parameters of T2* values. *p.adj < 0.05, **p.adj < 0.01, ***p.adj < 0.001.

Histograms of T2* value pixel distribution for a normal IVD (Pfirrmann grade I) and a degenerated IVD (Pfirrmann grade V) are presented in Figure 3. Figure 4 shows the T2‐HPs and T2*‐HPs of IVDs with increasing Pfirrmann grade. All the T2‐HPs and T2*‐HPs of IVDs were different among the different Pfirrmann grades (*p* < 0.001). For T2‐HPs, standard deviation (SD), 75th and 95th percentiles were all statistically different between adjacent Pfirrmann grades (*p*.adj < 0.05). Mean, median and entropy were statistically different between grade I and II, II and III, III and IV(*p*.adj < 0.05). Tenth and 25th percentiles were statistically different between grade I and II, II and III (*p*.adj < 0.05); skewness was statistically different between grade I and II, III and IV (*p*.adj < 0.05). Kurtosis was statistically different only between grade III and IV. For IVD T2*‐HPs, mean, SD, 75th and 95th percentiles were statistically different between grade I and II, II and III, III and IV(*p*.adj < 0.05). Median was statistically different between grade I and II, IV and V (*p*.adj < 0.05). Tenth and 25th percentiles were only statistically different between grade I and II (*p*.adj < 0.05). Skewness was statistically different between grade I and II, II and III, IV and V (*p*.adj < 0.05). Kurtosis and entropy were statistically different between grade II and III, III and IV (*p*.adj < 0.05).

Table 2 shows LFJ T2‐HPs and T2*‐HPs with Weishaupt grade. The mean and median of T2 values were statistically different between grade 0 and 1, 1 and 2 (*p*.adj < 0.05). Tenth, 25th percentile and Entropy of T2 values and were statistically different between grade 0 and 1 (*p*.adj < 0.05). No significant differences were found in other parameters among different Weishaupt grades.

**TABLE 2:** Histogram parameters of facet joints with different Weishaupt grades.

		Weishaupt 0	Weishaupt 1	Weishaupt 2	Weishaupt 3	Total
T2	Mean	65.98 ± 13.19	60.69 ± 14.06**	57.06 ± 12.92**	55.38 ± 12.50	59.24 ± 13.69
	SD	26.52 ± 10.11	23.92 ± 11.03	22.90 ± 10.33	23.81 ± 9.73	23.76 ± 10.58
	Median	59.03 ± 10.95	54.55 ± 12.12*	51.38 ± 10.81**	49.82 ± 11.18	53.26 ± 11.69
	10th	42.17 ± 7.35	39.87 ± 6.25*	39.05 ± 6.25	37.97 ± 6.47	39.57 ± 6.60
	25th	48.52 ± 8.48	45.59 ± 7.83*	44.57 ± 8.01	43.25 ± 7.71	45.23 ± 8.04
	75th	73.22 ± 15.94	68.24 ± 19.05	66.22 ± 18.00	64.17 ± 15.23	67.50 ± 18.12
	90th	102.05 ± 27.64	94.06 ± 32.95	90.21 ± 29.79	87.12 ± 25.46	92.60 ± 30.76
	Skewness	1.56 ± 0.68	1.67 ± 0.75	1.77 ± 0.82	1.84 ± 0.79	1.71 ± 0.78
	Kurtosis	2.89 ± 2.58	3.69 ± 3.56	4.21 ± 3.97	4.42 ± 3.70	3.89 ± 3.68
	Entropy	3.01 ± 0.24	2.92 ± 0.25*	2.93 ± 0.26	2.96 ± 0.27	2.93 ± 0.26
T2*	Mean	23.22 ± 7.91	23.62 ± 8.54	22.77 ± 9.73	21.07 ± 10.31	22.99 ± 9.17
	SD	11.32 ± 6.52	11.04 ± 7.09	10.74 ± 7.16	9.43 ± 7.02	10.78 ± 7.06
	Median	19.15 ± 6.64	19.52 ± 7.13	19.86 ± 10.33	18.70 ± 10.15	19.53 ± 8.77
	10th	12.48 ± 3.50	12.24 ± 3.25	12.84 ± 5.54	12.30 ± 3.65	12.50 ± 4.32
	25th	14.71 ± 4.44	14.41 ± 4.29	15.06 ± 7.02	14.27 ± 5.29	14.67 ± 5.59
	75th	26.45 ± 10.61	25.29 ± 13.86	25.73 ± 14.45	24.86 ± 15.52	25.53 ± 13.97
	90th	40.55 ± 22.83	35.49 ± 20.20	36.26 ± 21.34	33.57 ± 21.05	36.09 ± 21.04
	Skewness	1.97 ± 0.89	1.80 ± 1.04	1.76 ± 1.06	1.60 ± 1.09	1.78 ± 1.04
	Kurtosis	5.71 ± 5.08	5.19 ± 5.79	5.01 ± 6.01	4.47 ± 5.88	5.09 ± 5.82
	Entropy	2.68 ± 0.39	2.68 ± 0.37	2.65 ± 0.39	2.56 ± 0.40	2.66 ± 0.38

* *p*.adj < 0.05.

** *p*.adj < 0.01.

The results of the correlation analysis (Table 3) show that, except for lower percentiles (10th and 25th), the remaining IVD T2‐HPs and T2*‐HPs have a low to high correlation with the Pfirrman grade (|*r*| = 0.246–0.757, *p* < 0.05). No significant statistical correlation was observed between the LFJ T2‐HPs, T2*‐HPs and Weishaupt grading.

**TABLE 3:** Spearman coefficient test for the correlation between histogram‐derived parameters of IVDs and Pfirrmann grade.

		Correlation coefficient ***r***				
		L1/2 (*n* = 82)	L2/3 (*n* = 85)	L3/4 (*n* = 85)	L4/5 (*n* = 85)	L5/S1 (*n* = 83)
T2‐HPs of IVDs	Mean	−0.491***	−0.616***	−0.614***	−0.572***	−0.464***
	Median	−0.482***	−0.630***	−0.606***	−0.594***	−0.480***
	SD	−0.546***	−0.581***	−0.621***	−0.531***	−0.421***
	10th	−0.402***	−0.321**	−0.344**	−0.283**	−0.301**
	25th	−0.408***	−0.434***	−0.430***	−0.373***	−0.351**
	75th	−0.543***	−0.617***	−0.597***	−0.561***	−0.496***
	90th	−0.490***	−0.602***	−0.586***	−0.565***	−0.439***
	Skewness	0.302**	0.458***	0.259*	0.420***	0.382***
	Kurtosis	0.338**	0.462***	0.262*	0.376***	0.345***
	Entropy	−0.506***	−0.654***	−0.616***	−0.576***	−0.612***
T2*‐HPs of IVDs	Mean	−0.585***	−0.506***	−0.594***	−0.646***	−0.686***
	Median	−0.486***	−0.385***	−0.473***	−0.374***	−0.537***
	SD	−0.603***	−0.548***	−0.647***	−0.745***	−0.626***
	10th	−0.260*	−0.091	−0.096	−0.092	−0.214
	25th	−0.341**	−0.109	−0.243*	−0.195	−0.277*
	75th	−0.576***	−0.488***	−0.567***	−0.657***	−0.716***
	90th	−0.587***	−0.474***	−0.575***	−0.757***	−0.714***
	Skewness	0.588***	0.489***	0.575***	0.567***	0.520***
	Kurtosis	0.622***	0.535***	0.618***	0.688***	0.646***
	Entropy	−0.359***	−0.248*	−0.246*	−0.329**	−0.627***

Abbreviations: IVDs: intervertebral discs; LFJs: lumbar facet joints; T2*‐HPs: histogram‐derived parameters of T2* values; T2‐HPs: histogram‐derived parameters of T2 values.

* *p* < 0.05.

** *p* < 0.01.

*** *p* < 0.001.

**FIGURE 5:**
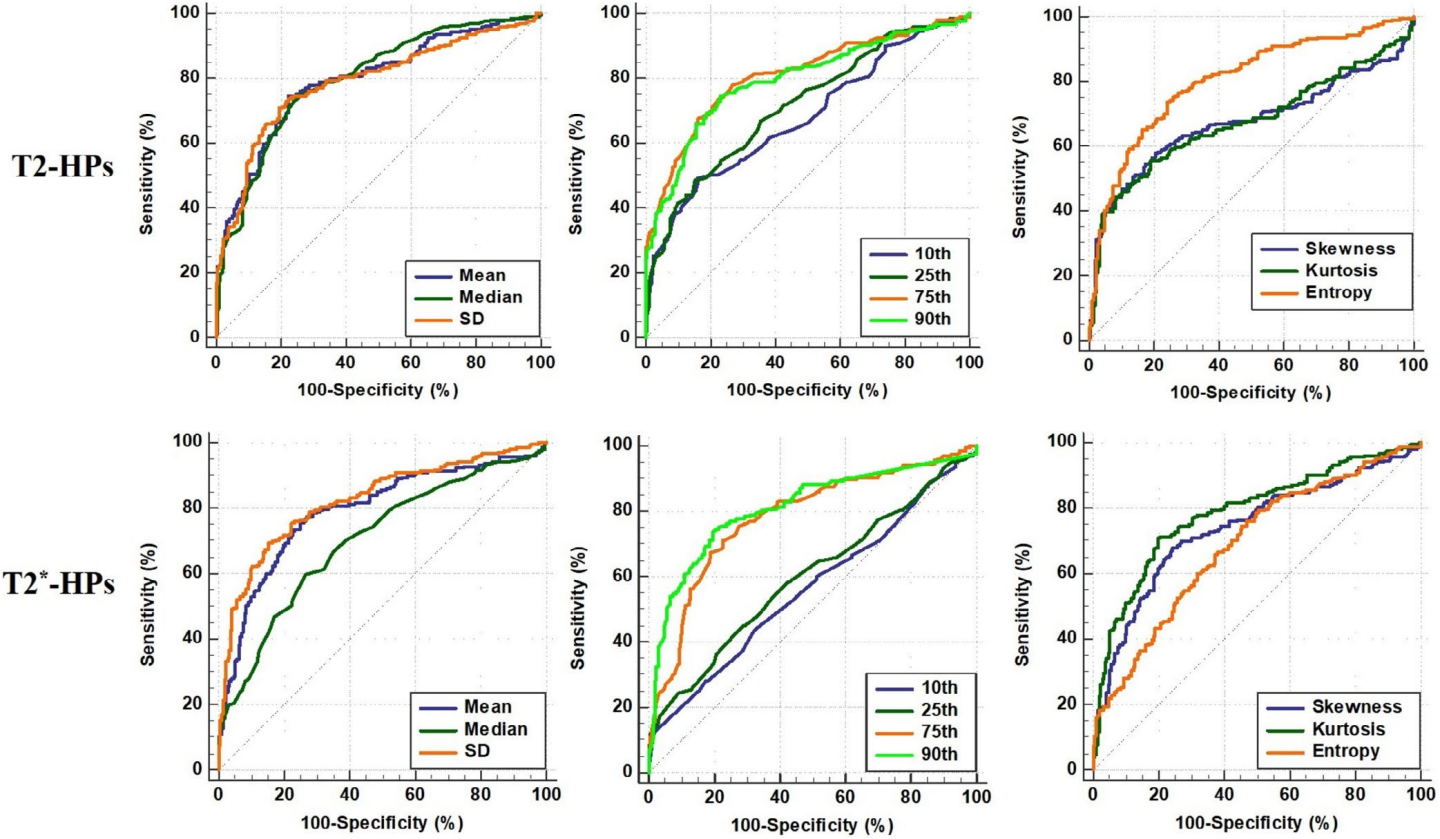
ROC analysis of T2‐HPs and T2*‐HPs for identifying normal (Pfirrmann grades I and II) and abnormal (grades III to V) IVDs.

Results from the ROC analysis are shown in Table 4 and Figure 5 which indicate the cut‐off values, sensitivity, and specificity of T2‐HPs and T2*‐HPs for identifying normal (Pfirrmann grades I and II) and abnormal (grades III to V) discs. All T2‐HPs and T2*‐HPs had the ability to distinguish normal IVDs from abnormal discs, with the AUC varying from 0.562 to 0.824.

**TABLE 4:** Receiver operating curve analysis of T2‐HPs and T2*‐HPs for identifying normal (Pfirrmann grade I and II) and abnormal (grade III–V) discs.

	T2‐HPs of IVDs				T2*‐HPs of IVDs			
	AUC	Cut‐off	Sensitivity	Specificity	AUC	Cut‐off	Sensitivity	Specificity
Mean	0.794***	67.60	74.64	77.73	0.792***	35.55	77.51	72.99
Median	0.797***	60.00	73.21	75.83	0.703***	25.00	59.81	73.46
SD	0.789***	29.44	73.68	77.73	0.824***	21.85	69.38	84.83
10th	0.679***	36.70	49.28	83.89	0.562***	12.95	44.02	67.30
25th	0.716***	43.50	48.80	84.36	0.599***	17.50	58.37	57.82
75th	0.809***	75.50	67.94	83.41	0.783***	40.00	71.29	81.25
90th	0.795***	105.00	74.64	76.78	0.807***	69.50	75.00	77.25
Skewness	0.673***	1.63	57.89	79.62	0.740***	1.50	67.94	75.83
Kurtosis	0.673***	3.38	55.50	81.04	0.788***	1.71	71.29	79.62
Entropy	0.802***	3.95	73.68	75.83	0.690***	3.88	76.08	53.08

Abbreviations: IVDs: intervertebral discs; T2*‐HPs: histogram‐derived parameters of T2* values; T2‐HPs: histogram‐derived parameters of T2 values.

*** *p* < 0.001.

Table 5 shows LFJ T2‐HPs and T2*‐HPs with Weishaupt grade. For T2‐HPs, only SD and Entropy cannot distinguish normal (Weishaupt grades 0 and 1) and abnormal (grades 2 and 3) LFJs, and all other parameters can distinguish them, with AUC changing from 0.551 to 0.615. For T2*‐HPs, only Mean and Entropy were reliable for identifying normal and abnormal LFJs with low AUC (0.572, 0.540, respectively).

**TABLE 5:** Receiver operating curve analysis of T2‐HPs and T2*‐HPs for identifying normal (Weishaupt grade 0 and 1) and abnormal (grade 2 and 3) facet joints.

	T2‐HPs of LFJs				T2*‐HPs of LFJs			
	AUC	Cut‐off	Sensitivity	Specificity	AUC	Cut‐off	Sensitivity	Specificity
Mean	0.604***	59.25	62.10	52.90	0.572***	19.64	50.86	62.41
Median	0.615***	54.42	69.92	48.72	0.527	17.00	49.39	55.68
SD	0.527	34.40	86.55	21.35	0.538	8.91	55.99	51.74
10th	0.567***	40.50	66.26	45.94	0.503	14.85	85.33	17.63
25th	0.569***	43.50	52.57	59.86	0.511	12.63	43.03	60.56
75th	0.555**	70.50	71.39	38.52	0.523	25.50	70.90	35.50
90th	0.547*	96.50	69.44	38.98	0.522	28.10	53.55	54.06
Skewness	0.555**	1.97	43.03	68.68	0.531	0.75	20.29	87.01
Kurtosis	0.551*	4.65	41.32	69.37	0.529	2.94	49.88	58.47
Entropy	0.501	2.92	47.19	56.84	0.540*	2.73	60.39	50.35

Abbreviations: LFJs: facet joints; T2*‐HPs: histogram‐derived parameters of T2* values; T2‐HPs: histogram‐derived parameters of T2 values.

* *p* < 0.05.

** *p* < 0.01.

*** *p* < 0.001.

### 3.5 Correlation Analysis Between LFJs and IVDs in the Three‐Joint Complex

Table 6 shows the qualitative information regarding discs and bilateral facet joint degeneration for all 420 lumbar three‐joint complexes according to Pfirrmann and Weishaupt grade. The correlation between IVD and LFJ degenerative grades was low across the five lumbar intervertebral levels (Right LFJ: *Kendall's tau‐b* = 0.384, 0.347, 0.227, 0.298, 0.271, *p* < 0.05; Left LFJ: *Kendall's tau‐b* = 0.284, 0.332, 0.359, 0.359, 0.252, *p* < 0.05). There was a moderate positive correlation between the grades of bilateral LFJ Weishaupt grades across the five levels (*Kendall's tau‐b* = 0.555, 0.462, 0.509, 0.587, 0.473, *p* < 0.05).

**TABLE 6:** Distribution of disc and bilateral facet joint degeneration of 420 three‐joint complexes.

IVD Pfirrmann grade	L‐LFJ Weishaupt grade				Total	R‐LFJ Weishaupt grade				Total

0	1	2	3		0	1	2	2	
I	11	42	12	2	67	17	36	13	1	67
II	19	61	57	7	144	11	78	48	7	144
III	9	33	48	17	107	8	45	44	10	107
IV	2	25	43	17	87	1	29	41	16	87
V	0	2	6	7	15	0	2	9	4	15
Total	41	163	166	50	420	37	190	155	38	420

Abbreviations: IVD: intervertebral disc; L‐LFJ: left lumbar facet joint; R‐LFJ: right lumbar facet joint.

**FIGURE 6:**
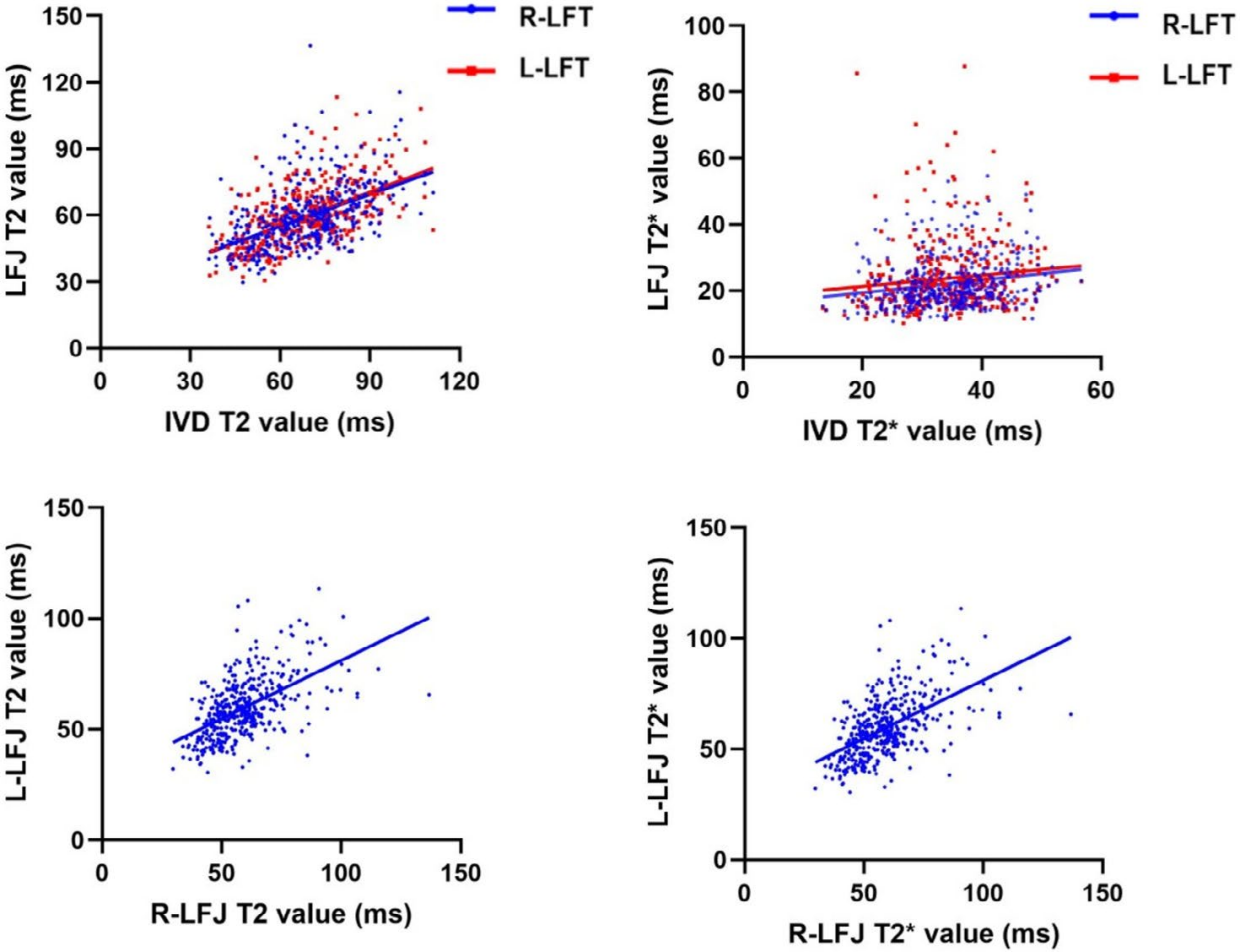
The scatter plots and fitting lines of T2 and T2* values for the anterior IVD and the posterior bilateral LFJs in the three‐joint complex.

Table 7 shows the results of the correlation analysis of the quantitative T2‐HPs and T2*‐HPs between the anterior IVD and the posterior bilateral LFJs in the three‐joint complex. Listed in the table are three representative intervertebral levels: L1/2 (cranial), L3/4 (middle), and L5/S1 (caudal). Mean, Median, SD, 10th, 25th, 75th and 90th of T2 were low or moderately correlated with those of the bilateral LFJs across all lumbar intervertebral levels (*r* = 0.275–0.606, *p* < 0.05), while the remaining parameters showed a low degree of correlation or no significant statistical correlation. Except for Skewness and Kurtosis, all the remaining parameters showed a low‐to‐high statistical correlation between the left and right LFJs (*r* = 0.283–0.728, *p* < 0.05). The absolute value of the correlation coefficient *r* between the bilateral LFJs was higher than that between IVD and LFJs in most cases. Figure 6 shows the scatter plots and fitting lines of the T2 and T2* mean values for the anterior IVD and the posterior bilateral LFJs in the three‐joint complex, with the fitting line between the paired bilateral LFJs being more inclined.

**TABLE 7:** The correlation analysis of the quantitative T2‐HPs and T2*‐HPs between the anterior IVD and the posterior bilateral LFJs in the three‐joint complex.

		Correlation Coefficient ***r***								
		IVD and L‐LFJ			IVD and R‐LFJ			L‐ and R‐LFJ		
		L1/2	L3/4	L5/S1	L1/2	L3/4	L5/S1	L1/2	L3/4	L5/S1
T2‐HPs	Mean	0.525*	0.532*	0.562*	0.449*	0.606*	0.559*	0.697*	0.479*	0.438*
	Median	0.551*	0.425*	0.489*	0.405*	0.575*	0.483*	0.684*	0.523*	0.541*
	SD	0.275*	0.421*	0.448*	0.286*	0.367*	0.367*	0.453*	0.356*	0.283*
	10th	0.467*	0.473*	0.451*	0.376*	0.483*	0.483*	0.623*	0.467*	0.470*
	25th	0.510*	0.481*	0.441*	0.385*	0.440*	0.511*	0.727*	0.437*	0.533*
	75th	0.321*	0.357*	0.363*	0.238*	0.426*	0.402*	0.728*	0.466*	0.329*
	90th	0.342*	0.412*	0.439*	0.333*	0.414*	0.426*	0.680*	0.466*	0.289*
	Skewness	−0.050	−0.008	0.162	−0.038	0.070	0.001	0.307*	0.388*	0.161
	Kurtosis	0.052	0.075	0.201	−0.109	0.130	0.013	0.281*	0.320*	0.131
	Entropy	0.106	0.108	−0.088	−0.035	0.261*	0.056	0.468*	0.472*	0.417*
T2*‐HPs	Mean	0.204	0.433*	0.234*	0.152	0.355*	0.387*	0.663*	0.734*	0.614*
	Median	0.111	0.323*	0.087	0.051	0.283*	0.236*	0.716*	0.783*	0.651*
	SD	0.293*	0.234*	0.211	0.317*	0.111	0.241*	0.564*	0.619*	0.363*
	10th	0.161	0.270*	0.201	0.098	0.203	0.330*	0.593*	0.517*	0.417*
	25th	0.147	0.305*	0.033	0.114	0.273*	0.247*	0.623*	0.645*	0.482*
	75th	0.157	0.201	0.070	0.088	0.239*	0.192	0.640*	0.631*	0.521*
	90th	0.220*	0.281*	0.168	0.222*	0.226*	0.222*	0.639*	0.647*	0.376*
	Skewness	−0.053	−0.125	−0.237*	−0.049	−0.009	−0.045	0.204	0.369*	0.240*
	Kurtosis	−0.081	−0.131	−0.281*	0.027	−0.006	−0.075	0.150	0.374*	0.141
	Entropy	−0.008	0.134	−0.041	0.013	0.146	0.112	0.597*	0.625*	0.656*

Abbreviations: IVD: intervertebral disc; L‐LFJ: left lumbar facet joint; R‐LFJ: right lumbar facet joint; T2*‐HPs: histogram‐derived parameters of T2* values; T2‐HPs: histogram‐derived parameters of T2 values.

* *p* < 0.05.

### 3.6 LBP Group vs. NC Group

The parameters of the IVD with the highest Pfirrmann grade were selected for comparison in each study subject. The Pfirrmann grading of 66 IVDs from the LBP group was as follows: 1 grade I, 10 grade II, 21 grade III, 27 grade IV and 7 grade V, with an average of 3.44 ± 0.93; the Pfirrmann grading of the 21 IVDs in the NC group was: 1 grade I, 8 grade II, 6 grade III and 6 grade IV, with a mean of 2.81 ± 0.93. There was a significant difference in Pfirrmann grading between the two groups (*p* = 0.012). All IVD T2‐HPs and T2*‐HPs were not significantly different between the two groups (*p* = 0.054–0.976).

**FIGURE 7:**
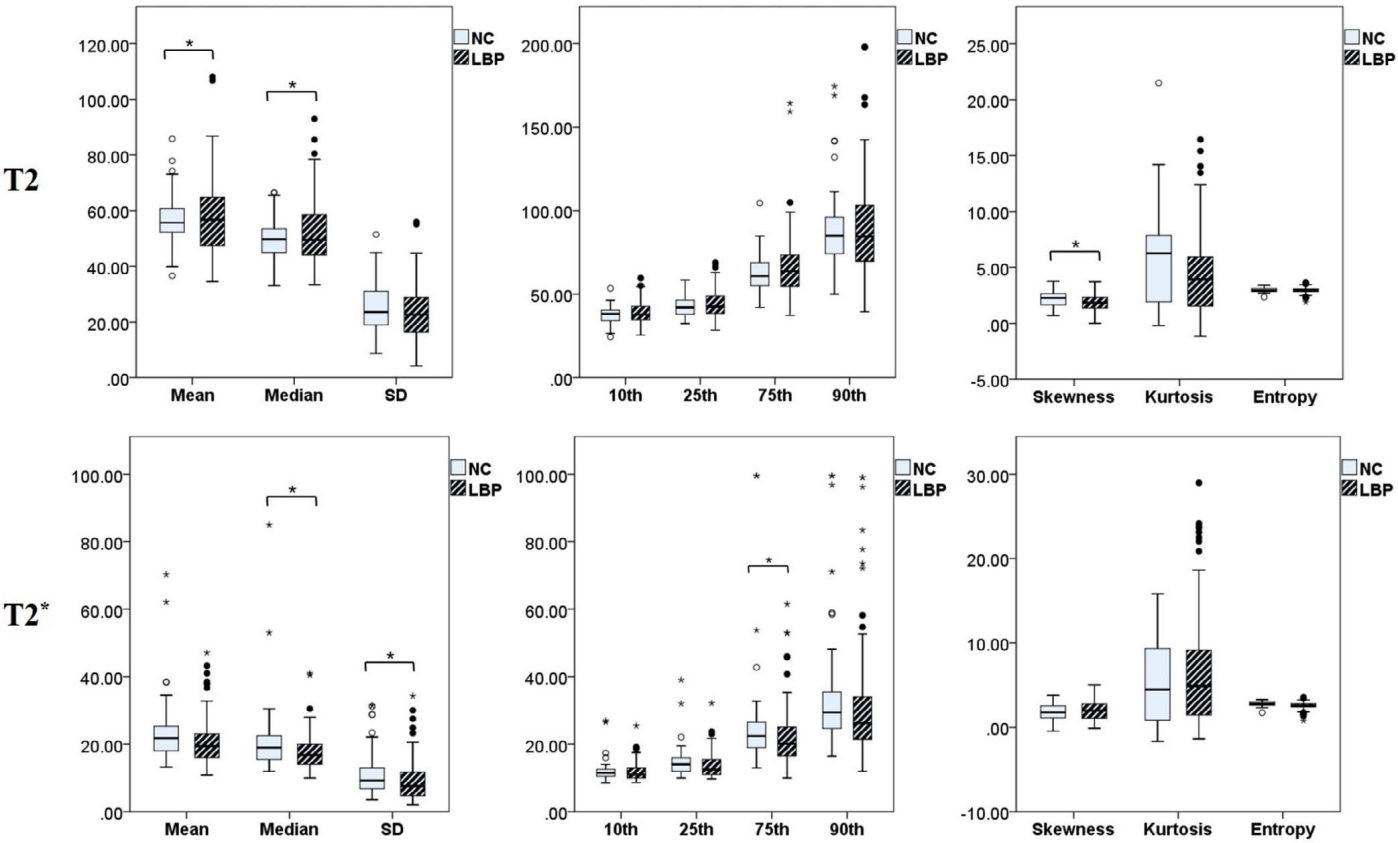
T2‐HPs and T2*‐HPs of facet joints between LBP group and NC group.

The parameters of the LFJs in pairs with the highest Weishaupt grade were selected for comparison in each individual. The Weishaupt grading of 132 LFJs from the LBP group was: 2 grade 0, 8 grade 1, 81 grade 2, 41 grade 3, with an average of 2.22 ± 0.62; the Weishaupt grading of 42 LFJs in the NC group was: 7 grade 1, 25 grade 2, 10 grade 3, with a mean of 2.07 ± 0.64. There was no significant difference in Weishaupt grade between the two groups (*p* = 0.152). Figure 7 shows that mean (*p* = 0.039), Median (*p* = 0.023), 25th percentile (*p* = 0.018), skewness (*p* = 0.014) of T2, and median (*p* = 0.017), SD (*p* = 0.039), 75th percentile (*p* = 0.016) of T2* were statistically different between LBP and NC groups; the remaining parameters were not significantly different between the two groups (*p* = 0.053–0.888).

## 4 Discussion

In this study, the interrelation between disc and facet joint degeneration was explored using morphological T2WI as well as biochemical T2 and T2* mapping. The feasibility of histogram analysis of T2 and T2* values on the degeneration of IVDs and LFJs was also assessed.

The results of this study show that the IVD and LFJ DI increases with age as widely agreed upon in the literature [15]. It is generally believed that lumbar degeneration begins in the IVDs followed by the LFJs [16, 17]. However, a histopathological study on 456 LFJs by Jun et al. [43] found that LFJ degeneration occurred as early as the age of 15 while the adjacent discs remained intact. The results of a recent animal experiment [44] showed that facet joint degeneration may be an initial process of cervical spine degeneration. The data presented in this study demonstrate that LFJ DI was higher than IVD at younger ages (20–39 years). These results challenge the commonly accepted paradigm of the degenerative process beginning at the IVDs and warn clinicians to pay attention to distinguish whether LBP comes from LFJ degeneration, especially young LBP patients with no other obvious spinal disorders. A report on 647 skeletal lumbar specimens also suggested LFJ degeneration appeared early, but with discs degenerating more rapidly and progressively than the LFJ [15]. Same as the previous trend, LFJ DI increased slowly with age in our study and only the amount of degeneration between the 40–49 and 50–59 year adjacent age groups was statistically significant. After the age of 40, the percentage of IVD degeneration was overwhelmingly greater, and the DI of the IVD increased precipitously. Therefore, we can only infer that LFJ degeneration occurs earlier and progresses slowly, while IVD degeneration progresses more rapidly, which requires longitudinal studies to confirm.

Previous studies have reported that the disc and the paired facets interact with each other during the spinal degeneration process, and that any abnormal changes in one part of the three‐joint complex affect the other two by impacting the spinal biomechanics [6, 45, 46]. Both qualitative and quantitative results of this study showed a higher correlation between the paired LFJs in the three‐joint complex than that between the disc and LFJs. We hypothesize that there is an interaction between the disc and the LFJs during degeneration, but the interaction is less intense than that between the posterior paired LFJs. From the perspective of histomorphology, the IVD is an independent fibrocartilage joint located at the triangular tip of the three‐joint complex, while the two LFJs are synovial joints, located on both sides of the symmetrical caudal triangles of the three‐joint complex; the paired LFJs are more functionally and structurally similar, and therefore may interact more closely with each other. Future studies will combine biomechanics to explore whether differences or asymmetries in the two LFJs affect IVD degeneration. The quantitative correlation coefficient *r* between the T2 and T2* values of intact IVD and LFJs in this study was slightly higher than that between nucleus pulposus (NP), annulus fibrosus (AF), and LFJs in previous reports [10, 11, 20]. No studies to the knowledge of the authors, have assessed the relationship between LFJ and the entire disc using qMRI.

Previous work [35, 36] suggested that histogram analysis of T2 mapping and conventional T2WI was feasible for classifying disc degeneration, but they used histogram indicators that were different from our study. The current study found that all T2 and T2* histogram parameters were capable of distinguishing discs with no or mild degeneration from abnormal discs with severe degeneration. For T2 and T2* values of discs, the higher percentiles (75th and 90th) often represent well‐hydrated zones with rich biochemical macromolecules such as proteoglycans, while the lower percentiles (10th and 25th) often reflect low hydration areas and areas of molecular disorder. In this study, higher percentiles were more reliable than lower percentiles in the establishment of the diagnosis of disc degeneration. This finding may reflect the pathological characteristics of disc degeneration in which biochemical molecules play a key role. Kurtosis represents the sharpness of a histogram peak. Skewness reflects the asymmetry of the observed value distribution with positive skewness indicating a longer right tail and larger absolute values indicating a larger deviation from the normal Gaussian distribution [47]. In this study, skewness and kurtosis increased with Pfirrmann grading, indicating that T2/T2* values of severely degenerative IVDs were concentrated on the left of the histogram. This result is consistent with the fact that the T2/T2* values of the NP decrease, and the boundary between the NP and the AF becomes blurred with degeneration. Entropy is an indicator of randomness and irregularity of the voxel distribution inside an ROI [28, 29, 31]. The current study shows reduced heterogeneity within severely degenerated IVDs. These histogram shape‐related parameters are independent of the absolute measure of T2/T2* values, so they are not affected by MRI scanners or sequence settings and have the potential to generate cut‐off values for the diagnosis of IVD degeneration if verified by large‐scale studies.

The diagnostic performance of histogram analysis on LFJ degeneration was poor. The correlation between T2‐HPs/T2*‐HPs and Weishaupt grade was statistically irrelevant in this study. No previous studies have evaluated histogram analyses of the LFJs. The complex structure of the facet joint, including its position below the median level of the disc, the curved surfaces especially in the lumbar region [48], and small size may contribute to these poor results. However, some LFJ T2‐HPs and T2*‐HPs (including the Mean, Median, 25th percentile, Skewness of T2 and Entropy of T2*) were significantly different between the LBP group and NC group, which has potential diagnostic value in distinguishing between LBP patients and healthy subjects. However, all IVD T2‐HPs and T2*‐HPs had no difference between the LBP group and NC group. Therefore, we speculate that facet joint lesions may be more closely related to LBP. More research is needed to explore and test these results in the future.

The major limitation of the present study was the utilization of the subjective morphological MRI grading systems for the LFJs and IVDs. The Weishaupt grading has moderate interobserver agreement for LFJ grading, and therefore may introduce bias. However, the Weishaupt grading is the only morphological scoring system of the facet joints with MRI. Therefore, the evaluation of the facet joint degeneration is difficult and a reliable grading system should be developed. The Pfirrmann grading system is a relatively reliable method for IVD grading with excellent interobserver agreement. However, both systems are based on the visual classification of imaging data which is inherently subjective without offering the reliability that histopathological examination can provide. Secondly, because of the three‐dimensional structure and small anatomical size of the facet joints, there are some errors in manual ROI delineation. Therefore, automatic methods will be needed to help it segment accurately in the future.

## 5 Conclusion

The current results showed that the DI of LFJ was higher than that of IVD under 39 years of age, but lower than IVD in the patients of 40–59 years old, which challenges the commonly accepted paradigm of the degenerative process beginning at the IVDs and warns clinicians to pay attention to distinguish whether LBP comes from LFJ degeneration. Both qualitative and quantitative results of this study showed a higher correlation between the paired LFJs in the three‐joint complex than that between the disc and LFJs, which provides a direction for exploring the biomechanical mechanism of spinal degeneration. Histogram‐derived parameters of T2 and T2* value correlated well with the Pfirrmann grading but not the Weishaupt grading, suggesting that histogram analysis of T2 and T2* values is feasible for detecting and grading IVD degeneration but the feasibility of histogram analysis of T2 and T2* values on grading LFJ is still controversial. Moreover, histogram analysis is expected to become a noninvasive tool that reflects the microstructure heterogeneity of degenerative lesions in vitro.

## Author Contributions

X.L. and X.L. take responsibility for the integrity of the work as a whole, from inception to finished article. X.L., J.M. and W.V.L. wrote and modified the manuscript. X.L., Y.L., B.H. and Y.X. collected data and performed statistical analysis. All authors read and approved the manuscript.

## Conflicts of Interest

The authors declare no conflicts of interest.

## Data Availability

The datasets generated and/or analyzed during the current study are available from the corresponding author on reasonable request.
